# Religious beliefs and practices in pregnancy and labour: an inductive qualitative study among post-partum women in Ghana

**DOI:** 10.1186/s12884-016-0920-1

**Published:** 2016-06-06

**Authors:** Lydia Aziato, Philippa N. A. Odai, Cephas N. Omenyo

**Affiliations:** Department of Adult Health, School of Nursing, College of Health Sciences, University of Ghana, Legon, Accra Ghana; College of Education, University of Ghana University of Ghana, Legon, Accra Ghana

**Keywords:** Spirituality, Phenomenology, Christianity, Prayer, Religious artefacts

## Abstract

**Background:**

Religiosity in health care delivery has attracted some attention in contemporary literature. The religious beliefs and practices of patients play an important role in the recovery of the patient. Pregnant women and women in labour exhibit their faith and use religious artefacts. This phenomenon is poorly understood in Ghana. The study sought to investigate the religious beliefs and practices of post-partum Ghanaian women.

**Methods:**

A descriptive phenomenological study was conducted inductively involving 13 women who were sampled purposively. Individual in-depth interviews were conducted in English, Ga, Twi and Ewe. The interviews were audio-taped and transcribed. Concurrent analysis was done employing the principles of content analysis. Ethical approval was obtained for the study and anonymity and confidentiality were ensured.

**Results:**

Themes generated revealed religious beliefs and practices such as prayer, singing, thanksgiving at church, fellowship and emotional support. Pastors’ spiritual interventions in pregnancy included prayer and revelations, reversing negative dreams, laying of hands and anointing women. Also, traditional beliefs and practices were food and water restrictions and tribal rituals. Religious artefacts used in pregnancy and labour were anointing oil, blessed water, sticker, blessed white handkerchief, blessed sand, Bible and Rosary. Family influence and secrecy were associated with the use of artefacts.

**Conclusions:**

Religiosity should be a key component of training health care professionals so that they can understand the religious needs of their clients and provide holistic care. We concluded that pregnant women and women in labour should be supported to exercise their religious beliefs and practices.

## Background

Religiosity and health are inter-related especially within the African context where illnesses have been linked to spiritual effects many years ago [1]. Witchcraft is associated with illness within the African context including issues of childbirth [2, 3]. Also, pregnancy and childbirth are associated with religious and traditional beliefs and practices in many countries [4–6]. In nursing and midwifery discourse, spirituality is an important component of care and should not be relegated to the background [7, 8]. It is an integrated part of the total care provided to clients and their families in all spheres of nursing and midwifery [9, 10].

During pregnancy, women intensify their prayers to God for protection, safe delivery and blessings [11]. Some women panic at the mention of caesarian section for fear of death during surgery and others who undergo caesarian section are stigmatized. This stigma transcends their generations [4]. Therefore, pregnant women would explore all spiritual and traditional options to ensure that they deliver spontaneously. Women commune with their God either individually or in a group. The prayer offered by pregnant women increases their faith and hope in God and it affords them the confidence of going through a safe delivery [11, 12].

The method of communication women use depends on the specific religious group the woman belongs to. Women may use religious artefacts such as blessed water and oil during prayers [13]. The blessed water and anointing/blessed olive oil are ordinary water and olive oil that the religious leader prays over. These artefacts may be used one-off or continuously for the duration of the pregnancy [14]. It is believed that the Holy Spirit (Spirit of God) uses the blessed water and oil as a medium to impact on the user [13]. During prayers some women also recite verses of the bible [11]. Some women also sing when communicating with their God and this may be considered a nuisance to others who do not use this form of communication with their God. Some religious denominations prescribe dressing mode for prayers especially at the place of congregation [15]. Others also remove their footwear before entering the prayer room. Religious restrictions pregnant women observe depend on the groups they belong to.

The majority (71.2 %) of Ghanaians are Christians [16] and the Pentecostal/Charismatic churches are fast growing churches in Ghana [17]. Within the Christian religion are leaders who contribute to the spirituality of women during pregnancy and labour. A growing phenomenon in Ghana is religious Pentecostal/Charismatic leaders praying for pregnant women and some giving the women religious artefacts such as anointing oil for their use. Some of these leaders also directly anoint the women and give them other spiritual directions concerning the use of artefacts or the performance of other specific activities aimed at safe delivery [18, 19].

Previous researchers have reported several traditional beliefs and practices associated with pregnancy, labour and the post-partum period. Some of these traditional beliefs and practices include food and water restrictions [6, 20, 21]; avoiding specific places such as the graveyard [22]; not going out at specific times in the day [5]; not associating with some people deemed to be evil [21] and drinking special herbal preparations [23]. Some women are restricted from work during pregnancy while others are not [24]. During labour, women suffer negative traditional beliefs that demand that they confess unfaithfulness to their partners when labour is delayed especially for those who deliver at home [4]. Specific dietary restrictions such as avoidance of fish in diet may predispose the pregnant woman to dietary deficiencies [20, 25–27].

The literature so far confirms that spirituality cannot be decoupled from pregnancy and child birth. However, there is little attention on Ghanaian women’s experiences of religious and traditional beliefs and practices in pregnancy and labour. This study sought to investigate post-partum women’s religious and traditional beliefs and practices during pregnancy and labour.

## Methods

### Design

The study adopted a descriptive phenomenological approach to investigate religious beliefs and practices in pregnancy and labour. An inductive approach was adopted to afford in-depth understanding of the phenomenon. A descriptive phenomenological study was considered appropriate for this study because it explores the personal experiences of women and thus Ghanaian women’s specific religious beliefs and practices were revealed [28].

### Setting

Post-partum women were recruited from the Korle-Bu Teaching Hospital (KBTH). The participants were resident in the Accra Metropolis and the hospital was a recruitment outlet for the study. Accra is the capital city of Ghana and the KBTH is the first tertiary health facility in Ghana. The maternity unit of the hospital has a post-natal clinic and potential participants were recruited from this clinic.

### Sampling and procedures of data collection

The study adopted a purposive sampling technique to recruit women who had delivered spontaneously within 2 months and were of the Christian faith. Women who had caesarian sections were excluded because the wider study from which this report was derived was focused on labour pain. Women who go through caesarian section may not go through labour pain before the surgery. Sample size was determined when no new finding was generated (saturation). Saturation was achieved with 13 women. Post-partum women were identified at the post-natal clinic. The place and the time of the interviews were at the convenience of the women. After the study had been explained to the women, they were allowed to make their own choices regarding participation. Those who consented to be part of the study were recruited. None of the women approached in this study refused to participate. A semi-structured interview guide was used to collect all the data in this study. The interview guide was developed based on the objectives of the study and probes were used to elicit further elaboration. Interviews were conducted in English, Twi, Ga and Ewe according to the participants’ preferences. Open-ended questions were asked to allow participants to express their thoughts and probes were used to follow-up on participants’ comments and emerging themes. The first author is experienced in qualitative interviewing and collected all the data. Individual interviews were audio-taped with a digital audio-recorder and later transcribed.

### Data management and analysis

Interviews conducted in English were transcribed verbatim and those conducted in Twi, Ga and Ewe were transcribed in English based on the meaning derived. These non-English transcripts were discussed with experts in these languages to ensure that participants’ comments were accurately represented. The transcripts were checked for accuracy and completeness by listening to the tapes and comparing them to the transcripts. Concurrent data analysis was undertaken in this study such that emerging findings were followed in subsequent interviews. The principles of content analysis were applied where transcripts were read several times and coded. Similar codes were grouped and re-grouped as the study progressed. Groups and sub-groups were named as themes and sub-themes according to the key findings [29]. Consistent with the tenets of phenomenology, the study held that knowledge is derived from the lived experiences of participants [28] and post-partum women have knowledge on their religious beliefs and practices. An inductive content analysis was therefore appropriate for data analysis. The research team discussed the themes and sub-themes to ensure that the women’s worlds were fully captured. The NVivo software version 10 was used to manage the data. The first author did the initial data analysis and the other co-authors coded independently and the themes and sub-themes were compared. Discrepancies in coding and theme development were discussed and a consensus was reached that best suited the data generated.

### Trustworthiness

Strategies adopted to ensure trustworthiness of this study included the use of the same interview guide and also one interviewer conducting all the interviews. Audit trail was kept for other researchers to verify the processes undertaken in this study. Verbatim quotes of participants’ comments were given to support the findings to allow for transferability of the findings in similar contexts. In-depth interviews allowed full exploration of religious beliefs and practices in pregnancy and labour. Concurrent data analysis ensured that participants’ comments were cross-checked in subsequent interviews and emerging themes were followed through member-checking. These approaches ensured validation of findings as the study progressed.

### Ethical considerations

Ethical clearance was obtained from the Noguchi Memorial Institute of Medical Research at the University of Ghana. Informed consent was obtained from each participant. Participants were given the option to withdraw from the study at any time. We ensured that no participant was stressed with provision of comfortable environment during the study and the babies of mothers were comfortable during the interview. Mothers were allowed to cuddle their babies and breast feed during the interview. Identification codes including PPW1 to PPW13 were assigned to the participants during data collection and these were used to present the findings. We ensured that comments on specific religious beliefs and practices of women were treated with respect and confidentiality. Data were kept under lock and key and soft copy of data were kept in a password protected computer.

## Results

### Demographic characteristics

The study involved 13 post-partum women who had delivered per vagina within the last two months. They were aged 18 to 35 years and were all Christians. With the exception of one participant, all the women were married.

### Description of themes and sub-themes

The themes were: Religious beliefs and practices (prayer, singing, thanksgiving at church, fellowship and emotional support); Pastors’ spiritual interventions in pregnancy (prayer and revelations, reversing negative dreams, laying of hands and anointing women); Traditional beliefs and practices (food and water restrictions, tribal rituals); Religious artefacts used in pregnancy and labour (anointing oil, sticker, blessed water, blessed white handkerchief, blessed sand, Bible and Rosary); family influence and secrecy.

Laying of hands involves the physical touch of pastors on women during prayer and in the process, they may anoint the women by applying anointing oil or blessed olive oil mostly on the head or forehead.

### Religious beliefs and practices

This theme describes sub-themes such as prayer, singing, thanksgiving at church, fellowship and emotional support during pregnancy and labour.

#### Prayer

The women in this study had a number of beliefs associated with pregnancy and delivery. The central belief was the likelihood of a negative outcome of pregnancy. Therefore, the women prayed to prevent any complications. They were of the view that pregnant women should be cautious as *extra forces* or evil spirits come to play during pregnancy. It was emphasized that pregnant women should pray.

*‘I know some pregnant women who did not pray or did anything spiritual; so, when they went to the labour ward, they came back alone and some died. Therefore, when you are pregnant, there are some extra forces that fight you in the spirit, and as such, you need to be cautious; extra cautious!’* (PPW8)

The women prayed believing that God will help them go through labour successfully and minimize their pain because the pain was unbearable. In those instances, the thoughts and concerns about the baby were not paramount. Some of the women prayed to God because they were not sure of their survival during labour.

*‘I was praying, at a point I just said God should bring out the baby and I will live; because, I didn’t know whether I will be able to survive the labour because the pain was very severe’.* (PPW12)

In addition, women prayed to God so they could deliver peacefully. *‘I prayed when I was at the hospital that God should be with me to enable me deliver in peace’* (PPW7). Also, prayer for women was done to ensure the protection from the blood of Jesus against accident or tragedy caused by the devil. The blood of Jesus was believed to be a potent protector although it was not seen physically. *‘…the pastors pray that the blood of Jesus should prevent all actions of the devil and when the time is right everyone should deliver safely; whatever tragedy or accident, God should prevent it*’ (PPW2). Pregnant women also prayed that God takes control of their delivery process when labour starts.

*‘I prayed to God to take away the human nature of the doctors and the nurses and to take absolute control over the whole delivery process’.* (PPW7)

Women in labour prayed that God will relieve them of severe labour pain. *‘I prayed that God should free me from the severe pain I was going through’.* (PPW12)

Some of the women who were initially scheduled for a caesarian section prayed for a normal delivery and they believed God answered their prayers when they did not undergo the surgery. The health care providers were *surprised* that they were delivered of their babies spontaneously.

*‘I was just thanking God and telling Him that He has done what I wanted for me. I was able to give birth myself without an operation. So when I was leaving the labour ward, the doctors and midwives were all surprised and even the doctor passed a comment that “Eii, someone that we were going to operate upon and in less than 30 minutes you have delivered safely” he was so surprised’.* (PPW3)

Some of the women prayed for themselves during pregnancy for safe delivery. The personal prayers were done at home or in the church.

*‘At times I wake up at dawn and before bed time and worship God on my own.* (PPW4); *‘when we had a programme at Church, I usually went and when they asked us to pray, I prayed about my safe delivery without anybody praying for me’.* (PPW3)

#### Singing

In addition to the prayer, some of the women sang quietly during labour and were asked by the midwives to keep quiet as they disturbed other women. During the singing, some women were thinking about the severe pain associated with the labour rather the unborn baby.

*‘a midwife warned me that I am not the only one on the ward; that I was making noise because I used to sing when praying; that was what I think made the noise’.* (PPW4)

*‘I tell God it is not by my strength but His; so, He should help me deliver safely. And I also sang quietly. …during the singing, I was thinking about the pain and how God was going to save me. …the pain was the first thing on my mind; I wanted the pain to reduce because it was unbearable. So at that time, seriously, I did not think much about the baby’.* (PPW8)

#### Thanksgiving at church

Following the thanksgiving at the labour ward, most of the women went to church to give testimonies for their safe delivery. *‘I went to give a testimony in church after my safe delivery’* (PPW2).

The women were either prayed for in the church or their family members prayed for them. Some of the women received instructions from those who prayed for them to ensure their safe pregnancy and delivery. Therefore the thanksgiving at church showed appreciation for the spiritual help received.

*‘…the pastors urged the rest of the congregation to assist us* (pregnant women) *in prayers for safe delivery’*. (PPW8)*; ‘…in my church, they call the pregnant women to the front to pray for them during church service. So I went to church to thank God and all those who helped me in prayer’.* (PPW2)

*‘…my sister was in a prayer force and they were praying for me. … so if I had to go somewhere, she told me to anoint yourself* (apply blessed olive oil on the body) *and I did. After the delivery, I went to church to God and the prayer force’.* (PPW8)

#### Fellowship and emotional support

Some of the women felt that it was *consoling* or reassuring to know that others are praying during pregnancy. This consolation is enhanced if it is accompanied with personal relationship with the pregnant women especially those having challenges with their pregnancies. In this instance, a participant was angry with her church when they did not show the needed concern during pregnancy.

*‘It’s consoling that somebody is praying for you, somebody is thinking about you and checking on you, I think it is really necessary. I was angry with my church when they didn’t check on me; they did not give me any attention when I was having challenges with my pregnancy; I thought they had deserted me and I was not happy’.* (PPW12)

### Pastors’ spiritual intervention in pregnancy

This theme describes the interventions received from pastors during pregnancy. Sub-themes such as prayer and revelations, reversing negative dreams, laying of hands and anointing women are described.

#### Prayer and revelations

Most of the women sought prayer support from pastors during pregnancy. The men of God had revelations about the pregnancy and prayed for the women. Some of these revelations related to witchcraft that aimed at a negative outcome of pregnancy.

*‘…the pastor said that someone from my paternal family wanted to give my child witchcraft so he prayed for me and sometimes when he saw any vision about me, he called me and prayed for me as the church members also prayed for me. He saw in a vision that the witches had planned to terminate my pregnancy so the pastor prayed to prevent it’* (PPW6)

In other circumstances, pastors were consulted when women could not feel foetal movements. The pastors in such situations revealed that the baby was *tied* in the womb after a hospital assessment showed a big baby. In addition to the prayers, a specific bible quotation was given to the women to use during prayers.

*‘…at 7 months I could not feel my baby move so when I went to the hospital and they said the baby was big that was why; so, I went to see my pastor and he prayed for me and told me that the baby had been tied up in my stomach’. He gave me a quotation that I should use to pray. …I cannot remember the quotation’*. (PPW2)

#### Reversing negative dreams

Other women had bad dreams during pregnancy and the pastors prayed with them even in the night. The pastors sometimes prayed in *tongues* (using different and unfamiliar language that is believed to be a gift of the Holy Spirit). The prayers were aimed at averting any negative consequences on the pregnancy.

*‘I had a dream that I had been delivered of my baby in the 7*^*th*^*month and someone took the baby away from me. … I told my pastor about the dream and he prayed for me. …he used to call me in the night; at times 12 midnight, 2 or 3 am to pray for me because he said that was when the witches are active. He prayed in tongues and I responded Amen! He said he saw in a vision that there was a plan to terminate my pregnancy so he prayed to prevent it’.* (PPW4)

#### Laying of hands and anointing women

In the quest to pray for the women for one reason or another, some pastors laid hands on the abdomen and applied blessed olive oil or anointing oil on it.

*‘The pastor asked me to lie down and he prayed for me and I used a cloth to wrap my lower abdomen and my abdomen was exposed. Then, he used the anointing oil on it. …since people were in the church, they were also praying and he poured some anointing oil in his hands and placed it on my abdomen and prayed’.* (PPW6)

In one instance, the pastor’s revelation showed that there was nothing wrong spiritually with the pregnancy. *‘…the pastor said there was nothing wrong’* (PPW2). However, a few of the women did not go to any pastor for prayers. ‘*…I never went to a pastor to pray for me when I was pregnant’.* (PPW3).

### Traditional beliefs and practices

This theme describes the traditional beliefs and practices women observed or undertook during pregnancy. Sub-themes of food and water restrictions and tribal rituals emerged. Women who reported traditional practices also went to church in addition to the traditional practices.

#### Food and water restrictions

Some traditional beliefs associated with pregnancy were that, pregnant women should not eat or drink in public to avoid effects of evil spirits.

*‘I was told not to eat or drink in public to avoid evil eyes; that was a traditional belief my mother told me so I was following it.…I wasn’t eating in public and I didn’t eat any food cooked by somebody; Apart from the one I cooked myself or maybe my mother or somebody closer to me cooked, I didn’t eat from any other source; even those on sale.* (PPW12).

#### Tribal rituals

A participant described vividly a traditional practice for first pregnancy that was mandatory for her tribe. She was camped for 3 days, tied, bathed with urine, and asked to buy shallots at dawn without talking to anyone. This ritual was necessary to ensure ancestral protection, a safe delivery and a normal baby.

*‘…a few days to my delivery, I was camped in a room for 3 days. During the period, I was tied with ropes and bathed with urine. On the third day, they brought me out early in the morning around 5:30 am and said I had to go and buy some small onions* (shallots) *and I was warned not to talk to anyone on my way. They gave me 3Gh Cedis in coins. Also, they said when I get to the market, I should not ask any questions; I should just put the money down and take the shallots; so, I did that and I returned and they said the onions were to be used immediately after I give birth. So, when I gave birth, they used it to prepare food for me the next morning. It is believed that if a woman does not do the ritual, the child will be abnormal. It is a traditional ritual that had been there for a long time …it is done for only the woman’s first pregnancy. …when they were doing it they called on the gods that if I have wronged anyone or someone has anything against me, my fore fathers or ancestors should intervene so that nothing bad happens to me. The ceremony was done by older women in my tribe’.* (PPW6)

### Religious artefacts used in pregnancy and labour

A number of religious artefacts were used by women to enhance their protection and ensure safe delivery. This theme describes the religious artefacts women used in pregnancy and labour such as anointing oil, blessed water, blessed white handkerchief, blessed sand, Bible and Rosary.

#### Anointing oil

Some women prayed over olive oil or anointing oil themselves and anointed themselves. The areas of the body anointed included the forehead, head, abdomen and feet. The word of God was added during the prayer over the oil perhaps to increase its potency.

*‘I just prayed over the anointing oil, believing that it works. I know that the anointing breaks the yoke of the devil; I have that faith. So sometimes I open the bible to support the anointing with a verse and I just anoint myself. …I apply it on my forehead and on my baby (tummy) or sometimes from my head to my feet or my feet alone’.* (PPW8)

Some of the women used anointing oil that was blessed by their pastors to prevent maternal and neonatal death. The use of the anointing oil was not regular because of lack of faith in it

*‘The pastor said I should use the anointing oil to smear my abdomen and that will ensure my normal delivery; if not, I will die with my baby. …though I didn’t believe in it, my husband did, so I did it for some time and I asked myself “what is the use of this?”. So, I stopped’.* (PPW12)

Some women were asked to add the anointing oil to their bathing water and use it for bathing.

*‘The pastor said I should pour some of the oil in the water I use to bath and then bath with it. I did it for about a week and I didn’t see any use of it, so I stopped’.* (PPW12)

#### Sticker

Women used stickers of pastors and churches during pregnancy and labour. Some women placed the sticker in the hair net they used to cover their hair on the labour ward while others rubbed it on the abdomen. Some women also placed the stickers in bottles of water they drank.

*‘a friend in labour had a sticker of her pastor in her hair net and after bathing, she would rub her belly with the sticker for a while and would then start smearing the anointing oil on her belly. …she also put one in bottled water and was drinking on the ward; I told her that the use of stickers alone without prayers cannot give her the desired spiritual protection’.* (PPW8)

#### Blessed water

Most women in this study used blessed water (water which had been prayed over by their pastors) as a religious artefact. The blessed water was sometimes used to wash the face. ‘*I used the blessed water to wash my face for a while and stopped’* (PPW13). Some women did not drink any ordinary water which may be bottled or in a sachet unless they had been blessed or prayed over by their pastors.

*‘I remember my first pregnancy, I wasn’t taking ordinary water, I bought a box of mineral water and we prayed over it before I drank it. I wasn’t allowed to take in any other water; …even if I was thirsty and you gave me water because my water was finished, I won’t drink it. I went home and took mine’* (PPW12)

#### Blessed white handkerchief

In addition, pregnant women were also given blessed white handkerchiefs to be placed under their pillows for protection. Some of the artefacts were used under the influence of mothers. Some husbands however were not aware of such artefacts.

*‘I also used a white handkerchief under my pillow; the pastor prayed over it and gave it to me to place under my pillow for protection’.* (PPW13)

#### Blessed sand

A woman reported using *sand* that was blessed by the pastor and the woman believed that it was safe to use. Water was added to the blessed sand and the solution was sieved and used for enema during pregnancy.

*‘The pastor blessed sand for me; he prayed over it for me to use …I had faith in it that when I use it, nothing will happen to me and it will work as the pastor said. He asked me to add water to the sand, sieve it and use it as enema from time to time and I did’.* (PPW2)

#### Bible and rosary

Some women used the Bible and rosary to pray during pregnancy. A few women did not believe in pastors praying for them. These artefacts were believed to provide protection for the pregnant woman. *‘I used just the Bible to pray; … I don’t believe in going to pastors to pray for me’* (PPW12). *‘…I used the rosary to pray; I had the rosary on my neck, I slept with it; I had the scapular too for protection’* (PPW11); *‘I usually used my rosary to pray’* (PPW13).

### Family influence and secrecy

Some pregnant women used anointing oil as a result of influence from their mothers although their husbands did not approve of such religious artefacts. A husband instructed that the woman should not allow the pastor lay hands on the abdomen during prayers. However, the woman on her own accord used the anointing oil on the abdomen in the morning, when going out and on the forehead for protection. The anointing oil was not sent to the labour ward because of the pain.

*‘My mum took me to a fellowship she worships with. My husband hates it when someone prays for you. He believes that you can pray for yourself and you will be saved because you do not know what power the pastor is using. So, when I went, he told me not to allow anybody to put the hand on my abdomen. The pastor gave me anointing oil that he had prayed over. When I wake up in the morning, I just apply some of the oil on my abdomen, before I go out and even on my forehead just to protect me; that was my belief. When I was going to the labour ward, I did not take the anointing oil with me because that time my mind was on the pain and I forgot’.* (PPW9)

In another instance, the woman applied the anointing oil on the abdomen to please the husband and did not use it when she was alone. *‘I wasn’t staying with my husband*; *so, when I went to his place, I tried to use the anointing oil but when I got to my station, I forgot about it’.* (PPW11)

*‘My husband never saw me use the blessed white handkerchief; …and I used the blessed water because I didn’t want to disobey my mother’.* (PPW13)

**Fig. 1:**
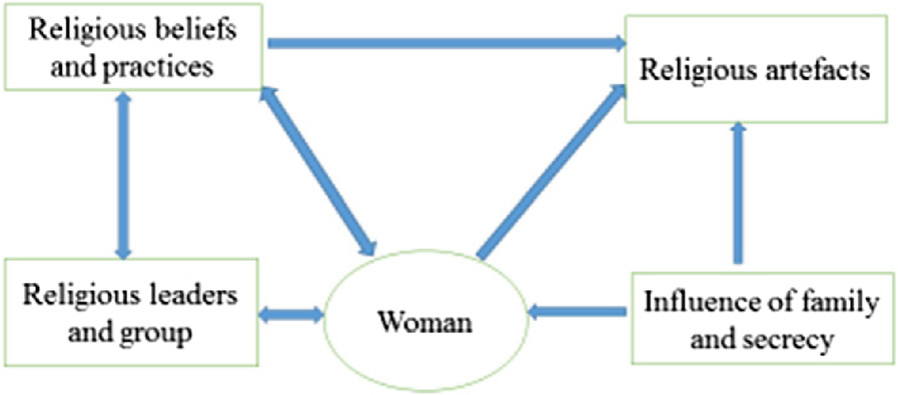
Religious beliefs and practices in pregnancy and labour

A framework that describes the themes is presented in Fig. 1: *Religious beliefs and practices in pregnancy and labour*.

## Discussion

This study investigated the beliefs and practices of Ghanaian women during pregnancy and labour with a focus on Christian women. A detailed insight was gained which showed that Ghanaian women prayed alone, in a group, and were prayed for either by family members or their religious leaders. Prayer was sometimes associated with singing and reciting the word of God. Women felt that group prayer and fellowship among members was reassuring and at the end of a safe delivery, women gave testimonies and thanks to God at Church. It was revealed that pastors prayed for women and provided some revelations that were directed at interventions for safe delivery. Pastors also prayed to reverse negative dreams, they laid hands on women and anointed them. A few of the participants reported their traditional beliefs and practices such as food and water restrictions and tribal rituals invoked extra protection from ancestors. An in-depth understanding of religious artefacts used in pregnancy and labour was gained. Women used anointing oil, blessed water, blessed white handkerchief, blessed sand, Bible and Rosary. Family members influenced some women to go for prayers and use religious artefacts during pregnancy and some used the artefacts secretly.

This study used a descriptive phenomenological approach that employed individual in-depth interviews to achieve a wealth of understanding on religious beliefs and practices in labour and pregnancy. Although the qualitative approach generated a deep insight into the phenomenon under study, the recruitment of only 13 women from an urban community who were Christians pre-supposes that the findings cannot be generalized to all pregnant women in Ghana. Perhaps rural women and those in other religious sets would have different experiences. However, verbatim quotations provided in this study would enhance application of findings to women with similar backgrounds.

The finding in this study that women prayed for God to ensure a successful delivery and prevent misfortunes or activities of evil spirits that affect the outcome of pregnancy suggests that women had faith in God. This assertion is supported by previous studies [30]. Women in this study implored the blood of Jesus on themselves to protect them from evil forces. The blood of Jesus is considered powerful and could prevent evil forces [31]. The prayer for God to take away the human nature of doctors and midwives suggests that women in this study believed that God can influence the doctors and midwives. The women also believed God could reduce labour pain when they prayed. It is important for health professionals to ensure adequate labour pain management so that the prayers of women can be answered. Praying in a group, individually and at different times of the day and singing and reciting the word of God [11] during prayers are characteristic of the Christian religion especially those of the Pentecostal/Charismatic denominations in Ghana. Fellowship among prayer group members was reported as reassuring and emotionally comforting [11, 19]. This suggests that religious groups should endeavour to give emotional support to pregnant women to enhance their mental state.

Most women in this study received prayer support from their pastors. The pastors gave some revelations that bothered around *witchcraft* and the condition of the baby and they prayed against any negative effect on the outcome of the pregnancy*.* Pastors prayed to reverse negative dreams and some prayed with women at night such as *2–3 am* on phone. Praying with pregnant women late at night may disturb their sleep and also contribute to conflict in their marriages if the spouses are also disturbed. In this light, although women would go to all lengths to ensure a safe delivery, there must be discretion when such prayer interventions are done. Pastors also anointed the abdomen of pregnant women by laying hands on the abdomen during prayers. The exposure of the abdomen in this study suggests that during prayer sessions where a part of the body is exposed, privacy should be ensured. Pastors praying for pregnant women have been reported by previous researchers [12].

The few women who reported traditional beliefs and practices such as not eating or not drinking outside the home supports previous studies and suggests that such restrictions may contribute to negative consequences for the woman and the unborn child [20, 26]. Tribal traditional practices for pregnant women that involve poor hygienic practices such as bathing the woman with urine may pose challenges for the woman. It implies that although ancestral protection is desired, the health and well-being of women should be taken into consideration during such rituals.

Religious artefacts used in this study included anointing oil, sticker, blessed water, blessed white handkerchief, blessed sand, Bible and Rosary. Praying over the anointing oil indicated dimensions such as self-prayer over the olive oil, pastors’ prayers and a family member praying over the olive oil before use. The anointing oil was applied on different parts of the body such as the abdomen, the forehead, the feet and the entire body. The belief that *the anointing breaks the yoke of the devil* meant that the anointing could destroy any negative influence of the devil. This finding is consistent with existing literature that indicates that the Holy Spirit works through the anointing oil [13]. The use of stickers of pastors or churches is a growing phenomenon in Ghana. However, women who add the sticker to their drinking water predispose themselves to health challenges as chemicals used to prepare the sticker could dissolve in the water. The use of blessed sand may also predispose women to worm infestations; thus, women should be cautious as they practice their faith. The use of blessed water has been reported by other researchers [13] and safe water is recommended for this purpose. Using blessed white handkerchief for protection appears to be an additional finding in this study although the Bible and Rosary have been used by women elsewhere [32].

The findings suggest that pregnant women deal with a lot of voices such as those of their pastors, mothers, husbands and health professionals. The fear of negative outcome of pregnancy and caesarian section compound the problem. Women then engage in religious practices to guarantee extra protection during pregnancy. In this regard, midwives and gynaecologists should understand that spirituality is an integral component of the care of pregnant women in Ghana and they should encourage and educate women to avoid the religious practices that could have negative health effects on them and the foetus. Individuality and right of choice should be ensured so that women would be allowed to demonstrate their religious beliefs and practices. This will ensure that the phenomenon of secrecy with the use of religious artefacts would be minimized.

Further studies could explore views of pastors who pray for pregnant women and those in labour to further understand the phenomenon under investigation. Perspectives of husbands and mothers can also be explored as well as those of women from other religious groups and rural areas to triangulate the findings in this study. The views of midwives and other health professionals who care for pregnant women could also be explored in future studies.

## Conclusion

Pregnancy and delivery have a strong religious connotation where a new life is born. The findings of this study give an in-depth insight into the religious beliefs and practices of women that suggest that some of the religious practices such as use of blessed sand and adding sticker to water could have negative effects. The secret use of religious artefacts pre-supposes that some women do not have the freedom to openly exercise their religious beliefs and practices even at home. Therefore women should be supported in their religious practices. Pastors who pray for pregnant women should be sensitive to their peculiar needs and context and revelations given to women should not lead to complications during pregnancy and labour.

## Abbreviation

KBTH, Korle-Bu Teaching Hospital
